# Knowledge of coronavirus disease 2019 (COVID-19) by medical personnel in a rural area of Thailand

**DOI:** 10.1017/ice.2020.159

**Published:** 2020-04-22

**Authors:** Patthamaporn Apaijitt, Viroj Wiwanitkit

**Affiliations:** 1 Nangron Hospital, Buriram, Thailand; 2 Dr DY Patil University, Pune, India; 3 Hainan Medical University, Haikou, China

*To the Editor*—Coronavirus disease (COVID-19) is a new respiratory infection that is a global public health problem; as of February 28, 2020, it had already caused disease in >60 countries. After it first appeared in China,^1^ Thailand became the second country where COVID-19 occurred.^2^ Presently, COVID-19 is under surveillance in Thailand. Even after several attempts to control the disease, both imported cases and local transmissions still occur.^3^ Based on the knowledge, attitude, practice (KAP) theory, good knowledge is necessary for successful disease control. Here, we report the results of a questionnaire on knowledge of COVID-19 administered to medical personnel in a rural area of Thailand. The setting is the Nang Rong district, a rural region of Thailand in Buriram Province, ~410 km from Bangkok and adjacent to Cambodia.

Briefly, a 10-question questionnaire (Table 1) was used to test the overall knowledge of 124 medical personnel (42 males and 82 females; average age, 36.7 ± 7.9 years) working in the study area (5 physicians, 81 nurses, 20 nurse assistants, 12 public health workers, and 6 other medical workers). The average total knowledge score was 6.26 ± 1.42. We observed no association between the total knowledge score and sex or age, but there was a significant association between total knowledge score and type of medical personnel. Many medical personnel still have a low level of overall knowledge about COVID-19, despite the emergence of the disease in Thailand and after several public health policies counteracting the outbreak have been implemented. Surprisingly, some physicians have a lower knowledge score than nonphysicians. These data indicate the necessity to improve education about the new disease among medical personnel. Medical personnel also educate the local population regarding disease and precautions, and if medical personnel are not knowledgeable, disease control may not succeed.

**Table 1.** Study Questionnaire

The sentence regarding COVID-19 is correct or not correct: 1. This disease is a respiratory disease. 2. Eating bats can cause this disease. 3. Soap can kill this pathogenic virus. 4. The incubation period of this disease is only 7 days. 5. Anyone who has not traveled to China has no risk for this disease. 6. All patients have fever. 7. All patients have a cough. 8. Some patients might have diarrhea. 9. Specific antiviral drugs are available. 10. A vaccine for prevention of this disease is available.
